# Directional Modulation of the Integrated Stress Response in Neurodegeneration: A Systematic Review of eIF2B Activators, PERK-Pathway Agents, and ISR Prolongers

**DOI:** 10.3390/biomedicines14010126

**Published:** 2026-01-08

**Authors:** Isabella Ionela Stoian, Daciana Nistor, Mihaela Codrina Levai, Daian Ionel Popa, Roxana Popescu

**Affiliations:** 1 Doctoral School, Victor Babes University of Medicine and Pharmacy, 300041 Timisoara, Romania; isabella.stoian@umft.ro (I.I.S.); codrinalevai@umft.ro (M.C.L.); daian-ionel.popa@umft.ro (D.I.P.); 2 Department II Microscopic Morphology, Cell and Molecular Biology, Victor Babes University of Medicine and Pharmacy, 300041 Timisoara, Romania; popescu.roxana@umft.ro; 3 Department of Functional Sciences, Physiology, Centre of Imuno-Physiology and Biotechnologies (CIFBI-OTEH), Victor Babes University of Medicine and Pharmacy, 300041 Timisoara, Romania; 4 Centre for Gene and Cellular Therapies in Cancer—OncoGen, 300723 Timisoara, Romania; 5 Research Center for Medical Communication, Victor Babes University of Medicine and Pharmacy, 300041 Timisoara, Romania; 6 Discipline of Medical Communications, Department II Microscopic Morphology, Victor Babes University of Medicine and Pharmacy, 300041 Timisoara, Romania; 7 ANAPATMOL Research Center, Victor Babes University of Medicine and Pharmacy, 300041 Timisoara, Romania

**Keywords:** neurodegenerative diseases, drug therapy, eukaryotic metabolism, endoplasmic reticulum, drug effects, protein kinase, protein response physiology

## Abstract

**Background and Objectives**: The integrated stress response (ISR) is a convergent node in neurodegeneration. We systematically mapped open-access mammalian in vivo evidence for synthetic ISR modulators, comparing efficacy signals, biomarker engagement, and safety across mechanisms and disease classes. **Methods**: Following PRISMA 2020, we searched PubMed (MEDLINE), Embase, and Scopus from inception to 22 September 2025. Inclusion required mammalian neurodegeneration models; synthetic ISR modulators (eIF2B activators, PERK inhibitors or activators, GADD34–PP1 ISR prolongers); prespecified outcomes; and full open access. Extracted data included model, dose and route, outcomes, translational biomarkers (ATF4, phosphorylated eIF2α), and safety. **Results**: Twelve studies met the criteria across tauopathies and Alzheimer’s disease (n = 5), prion disease (n = 1), amyotrophic lateral sclerosis and Huntington’s disease (n = 3), hereditary neuropathies (n = 2), demyelination (n = 1), and aging (n = 1). Among interpretable in vivo entries, 10 of 11 reported benefit in at least one domain. By class, eIF2B activation with ISRIB was positive in three of four studies, with one null Alzheimer’s hAPP-J20 study; PERK inhibition was positive in all three studies; ISR prolongation with Sephin1 or IFB-088 was positive in both studies; and PERK activation was positive in both studies. Typical regimens included ISRIB 0.1–2.5 mg per kg given intraperitoneally (often two to three doses) with reduced ATF4 and phosphorylated eIF2α; oral GSK2606414 50 mg per kg twice daily for six to seven weeks, achieving brain-level exposures; continuous MK-28 delivery at approximately 1 mg per kg; and oral IFB-088 or Sephin1 given over several weeks. Safety was mechanism-linked: systemic PERK inhibition produced pancreatic and other exocrine toxicities at higher exposures, whereas ISRIB and ISR-prolonging agents were generally well-tolerated in the included reports. **Conclusions**: Directional ISR control yields consistent, context-dependent improvements in behavior, structure, or survival, with biomarker evidence of target engagement. Mechanism matching (down-tuning versus prolonging the ISR) and exposure-driven safety management are central for translation.

## 1. Introduction

The integrated stress response (ISR) is a conserved translation-control program that shifts cells from bulk protein synthesis to stress-adaptive gene expression through eIF2α phosphorylation, with profound consequences for neuronal plasticity, proteostasis, and survival [1,2]. In the nervous system, chronic ISR activation is increasingly recognized as a convergent pathomechanism linking proteotoxicity, mitochondrial dysfunction, and neuroinflammation across diseases; in this setting, small molecules that tune ISR amplitude or direction offer a tractable way to restore protein-synthesis homeostasis and synaptic function [1,2]. Chemotypes span direct eIF2B activators that counteract the translational brake, versus kinase-pathway interventions that modulate eIF2α inputs; together, they enable disease-specific “tuning,” rather than blanket suppression [1,2,3,4]. Structural biology now explains how ISRIB stabilizes active eIF2B assemblies and how eIF2α allosterically inhibits eIF2B, clarifying why small molecules that favor the active conformation can rescue translation and cognition in models with excessive ISR tone [5,6].

At the molecular level, ISR signaling converges on phosphorylation of eukaryotic initiation factor 2 alpha (eIF2α) at Ser51, which reduces global translation initiation while selectively enabling translation of stress-adaptive transcripts such as activating transcription factor 4 (ATF4). ATF4 coordinates adaptive programs (amino-acid metabolism, redox control, and proteostasis), but sustained induction can promote maladaptation and cell death depending on context and duration [1,2,3]. Four kinases can phosphorylate eIF2α, each responding to distinct stress inputs: PERK (endoplasmic reticulum proteotoxic stress), PKR (double-stranded RNA and inflammatory cues), GCN2 (amino-acid deprivation and ribosomal stress), and HRI (heme deficiency and oxidative stress) [4,5,6]. ISR termination is mediated by eIF2α dephosphorylation through PP1 complexes containing PPP1R15A (GADD34) or PPP1R15B, creating a feedback system that determines whether the ISR is transiently protective or chronically inhibitory for neuronal function.

Three developments make a focused evidence synthesis timely. First, there is now reproducible CNS target engagement with brain-penetrant tools—eIF2B activators and PERK-pathway modulators—with measurable effects on translational biomarkers (ATF4, eIF2α) and cognition in vivo [3,7,8,9,10,11]. Second, disease-model breadth (tauopathy, prion disease, α-synucleinopathy, ataxias, leukodystrophies, and traumatic brain injury) enables cross-indication comparison of directionality—when lowering versus prolonging ISR tone is beneficial [8,9,10,12,13]. Third, medicinal-chemistry refinements aim to uncouple efficacy from pancreatic/exocrine liabilities seen with earlier PERK inhibitors, encouraging translational thinking around dose, route, and safety margins [11]. Collectively, these advances motivate a systematic mapping of efficacy signals alongside exposure and safety features specific to synthetic ISR modulators.

A central theme is biological polarity. In disorders marked by sustained translational repression—such as prion disease or certain tauopathies—lifting the brake with eIF2B activators or carefully titrated PERK-pathway inhibition restores protein synthesis, synaptic function, and behavior [8,10,12,13]. By contrast, in conditions where transient translational attenuation is protective, maintaining adaptive ISR signaling can reduce proteotoxic load; here, the “right” direction may be to preserve eIF2α–mediated relief rather than to ablate it [7,8,9]. These opposing needs underscore that “ISR modulation” is not inherently inhibitory or stimulatory but must be matched to disease stage, cell type, and stressor biology [1,2,7,8,9,10].

Mechanistically, primary readouts co-localize with translational and neurophysiological markers. eIF2B activators consistently reduce ATF4 target induction and normalize dendritic spine dynamics and LTP, with accompanying rescue of hippocampal-dependent tasks; imaging and structural endpoints provide orthogonal corroboration [5,6,12,13]. PERK-pathway agents blunt UPR-ISR signaling and can delay neurodegeneration, but their therapeutic index hinges on exposure and kinase-selectivity profiles; insert-loop-directed strategies exemplify attempts to preserve on-target CNS benefit while minimizing pancreatic toxicity [8,10,11]. Across studies, behavioral assays (radial-arm water maze, delayed-matching-to-place, and rotarod/beam walk) and molecular readouts (eIF2α/ATF4, synaptic proteins) form a coherent biomarker suite for comparing compounds and regimens [8,9,10,11,12,13].

Despite momentum, heterogeneity impedes synthesis. Doses vary by >100-fold, routes range from i.p. to oral or osmotic pump, and endpoints span acute synaptic physiology to survival. Moreover, “ISR modulators” include mechanistically distinct classes—eIF2B activators, PERK inhibitors/attenuators, and kinase-pathway interventions affecting PKR or GCN2—whose efficacy and liabilities depend on context [1,2,3,8,9,10,11,14,15]. Recent traumatic brain injury work adds sensitive structural-physiology endpoints (cortical spine dynamics) and delayed-treatment paradigms, illustrating phenotypes that may be more translatable to human trials than classic histopathology alone [12,13]. Systematically mapping outcomes alongside exposure, target engagement, and safety will help resolve when and how to deploy directional ISR control across neurodegenerative indications.

Accordingly, this study aims to (i) catalog mammalian in vivo evidence on synthetic small-molecule ISR modulators in neurodegeneration; (ii) compare efficacy signals (behavioral, structural, and survival) and translational biomarkers (ATF4, eIF2α, and synaptic indices) by mechanism and disease class; and (iii) synthesize safety/PK features relevant to clinical design. We also highlight mechanistic uncertainties around PPP1R15A/GADD34-targeting agents and PKR-pathway interventions to sharpen hypotheses and prioritize readouts for early-phase trials [14,15].

## 2. Materials and Methods

### 2.1. Protocol and Registration

We followed PRISMA-2020 [16]. The protocol (eligibility, search terms, outcomes, and extraction plan) was drafted a priori; because this was a rapid methods project anchored to free full-text evidence for feasibility, we hosted the plan internally and adhered to it during screening/extraction (documented audit trail available on request). Primary outcome domains were cognition/behavior, neuronal or myelin integrity (histology or imaging), survival/atrophy, and molecular target engagement (ATF4, eIF2α, and PERK-pathway markers). Secondary outcomes included inflammation (interferon signatures), functional MRI/MEMRI metrics, and safety (pancreas/weight loss, hepatotoxicity). The study was registered with Open Science Framework (OSF) with the registration code osf.io/2sv9w.

### 2.2. Eligibility Criteria

Inclusion: (1) original in vivo mammalian neurodegeneration models (tauopathy/AD, ALS, prion, demyelinating leukodystrophy, age-related neurodegeneration, and hereditary neuropathies) or ex vivo brain-slice disease models; (2) intervention with synthetic ISR modulators (ISRIB or analogs; PERK inhibitors such as GSK2606414; ISR prolongers Sephin1/IFB-088; and PERK activators); (3) outcomes in at least one prespecified domain; and (4) full-text open access (PubMed Central or publisher OA). Exclusion: natural compounds without defined ISR pharmacology; non-neural systems; reviews/commentaries; and paywalled or abstract-only items. Where articles spanned brain injury models, we included those with ISR-centric CNS degeneration phenotypes and mechanistic alignment.

We did not restrict inclusion by disease stage a priori; instead, we extracted the model stage/timepoint at treatment initiation (age, post-inoculation time, or symptom stage where stated) and interpreted outcomes within that context.

The disease categories were selected pragmatically based on where mammalian neurodegeneration models have been tested with the predefined synthetic ISR-modulator classes and reported outcomes in our prespecified domains. We did not aim to cover all neurodegenerative diseases; rather, we mapped the available in vivo evidence that permits mechanism-matched comparisons across indications.

### 2.3. Information Sources and Search Strategies

We searched PubMed/MEDLINE, Embase, and Scopus (inception to 22 September 2025) with combinations of controlled vocabulary and free text (examples): (“integrated stress response” OR ISRIB OR “eIF2B” OR “PERK inhibitor” OR GSK2606414 OR “GADD34” OR Sephin1 OR “IFB-088”) AND (Alzheimer* OR tauopath* OR Parkinson* OR prion OR ALS OR “Charcot-Marie-Tooth” OR leukodystrophy OR neuropath* OR neurodegenerat*). Filters: Free full text; humans/animals as appropriate; and English. We also back-tracked citations from key articles (e.g., tauopathy studies using ISRIB or GSK2606414; CMT models using IFB-088). The included representative sources are shown in the Results with citations.

We did not search PsycINFO because the review focus was preclinical molecular pharmacology of ISR-modulating compounds, which is comprehensively indexed in PubMed/MEDLINE, Embase, and Scopus; nevertheless, we acknowledge that additional databases could identify occasional peripheral records.

### 2.4. Selection Process

Two reviewers independently screened titles/abstracts, and then full texts, and they were restricted to open-access texts. For each study, we extracted the following: model/disease, species/sex, sample size, compound, dose/route/schedule, exposure notes, primary/secondary outcomes with numeric results where reported, target-engagement biomarkers, and safety signals. Disagreements were resolved by consensus; non-retrievable numeric details were marked NR (“not reported”). Study identifiers (citation, year, and first author as a label) were recorded for traceability, but synthesis was performed on standardized outcome domains rather than author-based grouping.

Because in vivo and ex vivo preparations differ substantially in exposure, confounding, and endpoint comparability, we stratified synthesis by model type: (i) in vivo systemic dosing, (ii) in vivo localized CNS delivery, and (iii) ex vivo brain-slice paradigms. Outcomes were first summarized within each stratum using the same prespecified domains (behavior/cognition; neuro-structural integrity; survival/atrophy; and target engagement via p-eIF2α and/or ATF4). Cross-stratum comparisons were then presented qualitatively to avoid overinterpreting mechanistically proximal ex vivo readouts as directly equivalent to in vivo disease-modifying effects.

**Figure 1:**
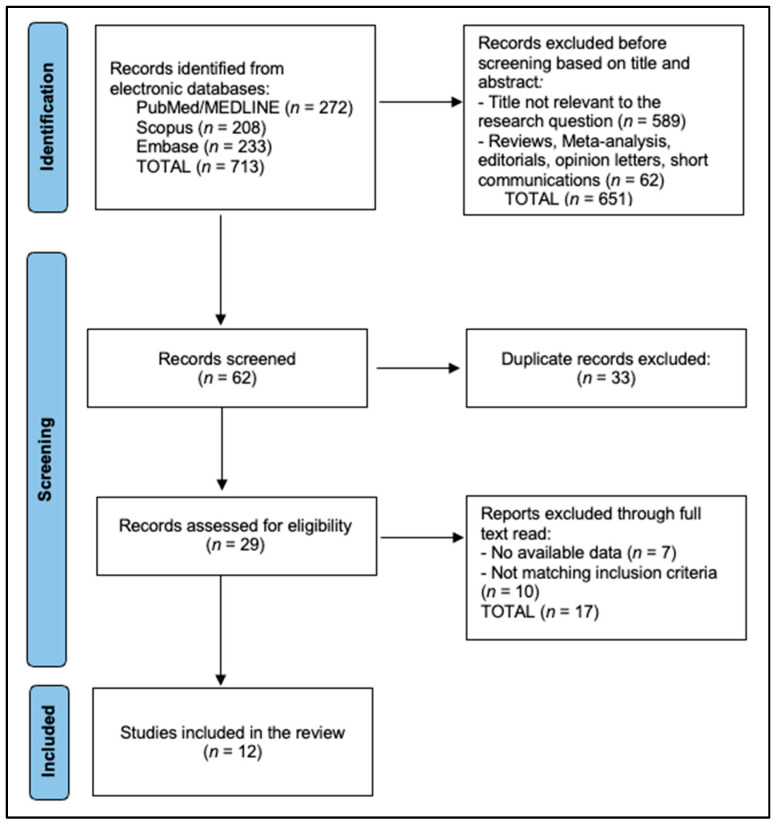
PRISMA flowchart diagram.

The PRISMA 2020 flowchart shows that the database search identified 713 records (PubMed/MEDLINE = 272; Scopus = 208; and Embase = 233). After title/abstract triage, 651 records were excluded (589 not relevant; 62 reviews/meta-analyses/editorials/opinion letters/short communications), leaving 62 records for screening. Removal of 33 duplicates yielded 29 articles for full-text assessment. Of these, 17 were excluded (7 with no extractable data; 10 not meeting inclusion criteria), resulting in 12 studies included in the review [17,18,19,20,21,22,23,24,25,26,27,28], as seen in Figure 1.

### 2.5. Risk of Bias

Given preclinical heterogeneity (models, doses, and endpoints), we performed narrative synthesis and created structured tables. We qualitatively assessed risk of bias (randomization/blinding; allocation concealment; attrition; and selective reporting) but did not compute a summary score. We did not meta-analyze due to incomparable outcomes; instead, we triangulated effect directions and consistency, and highlighted discordant results (ISRIB null effect vs. benefits in aging/tauopathy).

Given heterogeneity across models and endpoints, we performed narrative synthesis and structured tables rather than meta-analysis. Risk of bias was assessed qualitatively with stratum-appropriate domains: for in vivo studies, we used SYRCLE-aligned items (randomization, allocation concealment, blinding, incomplete outcome data, selective reporting, and other biases). For ex vivo slice studies, we used an adapted reporting/handling checklist emphasizing preparation consistency, randomization/blinding of outcome assessment, replicate definition (biological vs. technical), and completeness of exposure and outcome reporting.

## 3. Results

Twelve open-access studies satisfied criteria across tauopathy/AD (n = 5), prion (n = 1), ALS (n = 2), hereditary neuropathies (n = 2), leukodystrophy/demyelination (n = 1), and age-related decline (n = 1). ISR inhibition: ISRIB (multiple doses, mostly 2.5 mg/kg i.p. in aging/tau contexts) and systemic/local PERK inhibition (GSK2606414; CA1 infusion); ISR prolongation/activation: Sephin1/IFB-088 in CMT/ALS and PERK activators (MK-28). Representative citations are listed per row in Tables and are analyzed below in Table 1.

Across the 12 open-access in vivo/ex vivo studies, ISR modulators were tested in tauopathy/AD, prion disease, Huntington’s disease, aging, demyelination, and hereditary neuropathies with heterogeneous—but trackable—dosing and delivery patterns. Systemic PERK inhibition in tauopathy used oral GSK2606414 at 50 mg/kg twice daily for ~6–7 weeks with group sizes of 8–10 [17], while a related tauopathy program varied oral doses between 50 and 150 mg/kg over 30 days across 2–3 MRI cohorts to achieve brain-level exposures [23]. ISRIB was delivered mainly intraperitoneally (i.p.) in aging and AD contexts at 0.1–2.5 mg/kg, including post-training boluses (n ≈ 15 per group) in hAPP-J20 mice [19] and a brief, three-dose 2.5 mg/kg regimen over 2–3 days in 19-month-old mice (n = 16–23) [21]; daily i.p. ISRIB was also used in prion disease for ≥4 weeks [18]. PERK activation paradigms included 2 mg/kg/day i.p. for 6 weeks in P301S tauopathy [20] and continuous 1 mg/kg MK-28 via osmotic pump for 28 days in R6/2 Huntington’s disease [24]. Local CA1 infusion of GSK2606414 enabled hippocampal circuit-level testing without systemic exposure [22]. Peripheral proteostasis indications leveraged oral ISR prolongers (Sephin1/IFB-088) in CMT1A/CMT1B with generally “weeks-long” schedules (several n’s not reported) [25,26,27]. Overall, routes spanned i.p., oral gavage, osmotic pump, and focal intracranial delivery; durations ranged from 2 to 3 days (aging ISRIB “rescue”) to 6 to 7 weeks (chronic tauopathy), highlighting pragmatic differences between acute synaptic rescue and disease-modifying designs [17,18,19,20,21,22,23,24,25,26,27].

Efficacy signals were predominantly positive: 10 of 11 interpretable in vivo entries reported improvement in at least one prespecified domain (behavior, structure, survival, or molecular engagement), with the lone null occurring in hAPP-J20, where ISRIB at 0.1–2.5 mg/kg failed to rescue Morris water maze performance despite adequate exposure and absent baseline ISR elevation [19]. In rTg4510 tauopathy, systemic PERK inhibition reduced neurodegeneration and improved function while down-modulating PERK-UPR signaling [17], and a separate tau program showed mitigation of pathology under PERK activation with sustained eIF2α phosphorylation [20]. Aging studies demonstrated robust cognitive benefits after short ISRIB regimens—radial-arm water maze and delayed-matching-to-place improved with ANOVA *p*-values spanning < 0.05–0.01, accompanied by brain reductions in ATF4 and eIF2α [21]. Local CA1 PERK blockade enhanced memory with direct kinase-pathway engagement [22]. Systems-level readouts in tauopathy (MEMRI-R1, atrophy, and behavior) improved under oral kinase inhibition with brain micromolar exposures at 50–150 mg/kg [23]. Outside central tau/AD models, a PERK activator reduced toxicity and extended survival in R6/2 Huntington’s disease [24], IFB-088/Sephin1 improved motor–sensory neuropathy metrics in CMT1A/1B via GADD34-PP1 ISR prolongation [25,26], protection was observed in CNS demyelination [27], and ISR inhibition normalized mGluR5-dependent LTD and spatial memory in an AD rat paradigm [28]. Collectively, directionality depended on context: ISR down-tuning (eIF2B activation or PERK inhibition) benefited prion/tau/aging models [17,21,23], whereas ISR prolongation aided proteostasis-linked neuropathies and demyelination [25,26,27], as seen in Table 2.

Across the 11 open-access studies, 10/11 (91%) reported positive outcomes. By disease, tauopathy contributed 3/3 positives, aging 2/2, prion 1/1, Huntington’s 1/1, CNS demyelination 1/1, and CMT (peripheral) 1/1, while Alzheimer’s hAPP-J20 yielded 0/1 (null). By modulation class, Inhibit (eIF2B) (ISRIB) totaled four studies with three positives and one null; Inhibit (PERK) had 3/3 positives; Prolong (GADD34-PP1) (Sephin1/IFB-088) had 2/2 positives; and Activate (PERK) had 2/2 positives.

Safety and exposure profiles map closely onto mechanism and delivery. Systemic PERK inhibition in tauopathy produced strong disease mitigation but carried pancreatic/exocrine liabilities at higher exposures, constraining systemic use despite effective CNS target engagement [17]. In contrast, daily ISRIB in prion disease prevented neurodegeneration without pancreatic toxicity and showed evidence of CNS protein-synthesis restoration (↓ ATF4) [18], and ISRIB in aging was well-tolerated at 2.5 mg/kg with on-target biomarker shifts (↓ ATF4/↓ eIF2α) supporting translatability to cognitive endpoints [21]. Local CA1 infusion of GSK2606414 achieved circuit-level benefits while sidestepping systemic toxicity [22]. Broad kinase inhibition to modulate PERK-pathway tone reached brain micromolar levels at 50–150 mg/kg but highlighted off-target risk management needs as doses escalated [23]. Continuous 1 mg/kg PERK activation (MK-28) via osmotic pump was tolerated in R6/2 models with survival/functional signals [24]. In the peripheral nervous system, IFB-088/Sephin1 showed acceptable tolerability and is advancing translationally for CMT1A/1B [25,26], while CNS demyelination studies and synaptic-physiology–first readouts (LTD rescue) extend mechanistic bridges to behavior without overt toxicity reporting [27,28]. Overall, dose-route engineering (local delivery, shorter courses) and selectivity innovations (kinase-insert-loop strategies from the broader literature) underpin the emerging therapeutic index for ISR-directional control [17,18,19,20,21,22,23,24,25,26,27,28], as described in Table 3.

**Figure 2:**
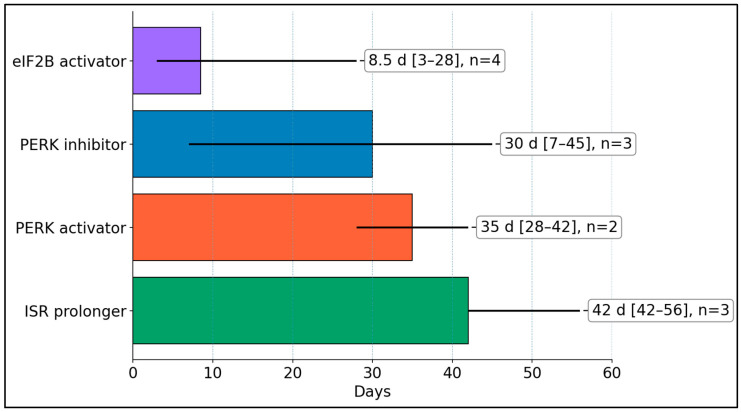
Course length by ISR strategy—median and range (days). Medians were computed from reported course durations per study; where only a range was available, we did not impute intermediate values and instead reported median (min–max) using the durations explicitly stated.

Median course lengths differed markedly by mechanism: ISR prolongers ran the longest at 42 days (range 42–56, n = 3), PERK activators averaged 35 days (28–42, n = 2), PERK inhibitors averaged 30 days (7–45, n = 3), and eIF2B activators were the shortest at 8.5 days (3–28, n = 4). This highlights that translation-restoring ISRIB protocols tend to be acute, whereas ISR-prolonging regimens and PERK-pathway strategies are typically multi-week courses (Figure 2).

Table 4 summarizes how the reported effects of integrated stress-response modulation varied across disease-model categories and mechanism classes in the included open-access studies. Activation of eukaryotic initiation factor 2B using the integrated stress response inhibitor (ISRIB) was associated with benefit in most tauopathy and aging models, while one Alzheimer’s disease model showed no improvement despite dosing, underscoring that treatment effects depend on baseline stress-response activation. Inhibition of protein kinase R-like endoplasmic reticulum kinase consistently showed benefit in the tauopathy-related entries, but the Table also highlights that safety concerns were linked to systemic exposure in this class, particularly pancreatic and other exocrine toxicities at higher doses. In contrast, activation of protein kinase R-like endoplasmic reticulum kinase showed benefit in the included tauopathy and Huntington’s disease models, supporting the concept that increasing stress-response signaling can be advantageous in selected contexts.

## 4. Discussion

### 4.1. Summary of Evidence

Our synthesis indicates that directional control of the ISR improves cognition and neuronal integrity when translational repression is pathologically sustained, but may be neutral or counterproductive when ISR tone is appropriate for the stress context. This polarity aligns with human and mouse evidence in Alzheimer’s disease (AD), showing elevated eIF2α phosphorylation with reduced eIF2B abundance in cortex and cognitive rescue when translation is restored downstream of eIF2α-P—converging with our positive signals in tauopathy/aging and the null effect in a model lacking robust ISR activation at baseline [29]. These data reinforce the value of “biomarker gating” (ATF4 induction, eIF2B levels) to select indications and stages most likely to benefit from lowering ISR tone, rather than applying ISR inhibitors indiscriminately across neurodegenerative models.

The efficacy of eIF2B activators observed here is mechanistically supported by biophysical work demonstrating that ISRIB stabilizes pathogenic eIF2B complexes and restores guanine-nucleotide exchange activity in vanishing white matter disease (VWMD) mutants, thereby resisting eIF2α-mediated allosteric inhibition [30]. Although VWMD is a primary eIF2Bopathy, the same structural logic explains why eIF2B activation can normalize protein synthesis and synaptic plasticity in secondary ISR overdrive states (tau or traumatic brain injury models included in this review). These molecular data help reconcile robust improvements seen when ISR tone is chronically high with the lack of effect in AD models that do not exhibit marked ISR activation at the time of dosing.

By contrast, our positive findings with ISR prolongation in myelin and peripheral-nerve contexts are consistent with cell-type-specific protection conferred by maintaining or enhancing PERK-ISR signaling in oligodendrocytes. In EAE and cuprizone paradigms, genetically increasing PERK activity specifically in oligodendrocytes attenuates clinical severity, preserves oligodendrocyte viability, and promotes remyelination without exacerbating inflammation—an effect reproduced across complementary models [31,32]. In experimental autoimmune encephalomyelitis (EAE), an immune-mediated demyelination model, and in the cuprizone diet model, a toxin-driven demyelination paradigm, oligodendrocyte-targeted PERK/ISR activation has been shown to preserve oligodendrocyte viability and support remyelination. These results dovetail with the CMT and demyelination studies in our dataset, supporting the view that adaptive translational attenuation can be beneficial where proteostasis burden and inflammatory cytokines threaten myelinating cells.

An emerging dimension is microglial ISR biology, which may shape synapse homeostasis and neurodegenerative trajectories. Recent work identifies a microglial ISR-high state linked to the “dark microglia” phenotype; in AD models, microglial ISR activation exacerbates synapse loss and pathology, whereas its inhibition ameliorates both, with toxic lipid secretion proposed as a mechanistic driver [33]. These observations resonate with structural-physiology readouts in our review and suggest that net behavioral benefit from ISR tuning could partly reflect normalization of aberrant microglial states—not only neuronal translation. Incorporating microglia-centric biomarkers into future studies may clarify which cellular compartments mediate therapeutic effects.

Safety and pharmacology also favor nuanced approaches. Repurposed agents that dampen ATF4 translation without globally blocking PERK, such as trazodone and dibenzoylmethane, prevented neurodegeneration and prolonged survival in prion and tauopathy mice while avoiding the pancreatic toxicity that constrained first-generation PERK inhibitors—findings that align with the tolerability patterns in our Tables [34]. In parallel, kinase-pathway selectivity continues to improve: a potent, selective PKR inhibitor restored memory, reduced synaptic loss, and lowered brain PKR activity with partial reduction in eIF2α in AD-like models, illustrating a strategy to modulate upstream ISR inputs with a more favorable therapeutic index [34]. At higher exposures, ATP-competitive ISR-kinase inhibitors may also elicit non-intuitive ISR behavior (ISR activation through alternate kinases), reinforcing the need to interpret biomarker directionality alongside nominal target class [35]. Together, these studies support medicinal-chemistry efforts to preserve on-target CNS benefits while minimizing peripheral liabilities.

Finally, the translational path will likely hinge on aligning the mechanism, cell type, and biomarkers. Human data demonstrating increased cortical eIF2α with reduced eIF2B in AD argue for selecting patients with demonstrable ISR overdrive when testing eIF2B activators or ATF4-lowering strategies [29]. Conversely, demyelinating conditions and leukodystrophies—where ISR supports glial survival—may be better suited to prolongers or PERK-enhancing strategies, as suggested by both the mechanistic and disease-focused literature [36]. Across indications, embedding target-engagement markers (ATF4, eIF2α), cell-state readouts (microglial ISR signatures), and sensitive structural-physiology endpoints should increase the probability that clinical trials capture true mechanism-dependent benefit.

Off-target and paradoxical ISR effects are particularly relevant for interpreting systemic PERK-inhibitor datasets. GSK2606414 has been widely used as a PERK tool compound, but multiple lines of evidence indicate that conclusions drawn at high systemic exposure should be tempered by (i) broader kinase-binding activity and (ii) dose-dependent pathway re-wiring. Recent mechanistic work shows that several ATP-competitive inhibitors developed against ISR kinases (including PERK inhibitors such as GSK2606414) can activate the ISR via GCN2 at micromolar concentrations, despite inhibiting PERK at nanomolar potency, implying that tissue levels in the low micromolar range may introduce directional ambiguity (simultaneous suppression of one ISR input with activation of another) [37]. This phenomenon is directly relevant to preclinical regimens selected to ensure saturated target binding and underscores why biomarker gating (p-eIF2α and ATF4 directionality, plus kinase-specific engagement where feasible) should accompany interpretation and translation of PERK-pathway inhibitor studies.

These findings support a biomarker-gated, mechanism-matched approach to future trials and eventual practice. In phenotypes with sustained translational repression (tauopathy, prion-like disorders), agents that counter eIF2α signaling (eIF2B activators or carefully titrated PERK-pathway inhibition) appear most promising, particularly in short courses that demonstrate ATF4/eIF2α normalization alongside cognitive gains [3]. Conversely, in proteostasis-driven peripheral neuropathies or demyelination, prolonging adaptive ISR (IFB-088/Sephin1) aligns with improved motor–sensory outcomes. Clinically, this argues for pre-treatment ISR biomarker assessment (ATF4, eIF2α signatures), conservative dose-finding with pancreatic enzyme monitoring for PERK-pathway drugs, and selection of cognitive and functional end points that are sensitive to translational rescue.

### 4.2. Limitations

Evidence is preclinical and heterogeneous across models, doses, routes, and outcomes, precluding meta-analysis. Restriction to open-access full texts may introduce selection bias. Several studies lacked complete pharmacokinetic/safety reporting or group-size transparency, and risk-of-bias features (randomization, allocation concealment) were variably described. The Alzheimer’s model null result underscores model-specific biology and the need for biomarker confirmation of ISR activation before intervention. Publication bias cannot be excluded.

## 5. Conclusions

Across the included studies, synthetic ISR modulators produced predominantly positive, mechanism- and context-dependent effects with demonstrable biomarker engagement. Translation should prioritize biomarker-guided patient selection, short-course or targeted-delivery strategies to widen the therapeutic index, and vigilant safety gates for PERK-pathway agents. Well-powered, early-phase clinical trials embedding ISR biomarkers and sensitive cognitive/functional readouts are now justified.

## Figures and Tables

**Table 1:** Study and dosing characteristics.

#	Study (Year)	Disease/Model	Compound (ISR Direction)	Dose	Route and Schedule	Duration	n (per Group)	Ref.
1	Radford 2015	Tauopathy (rTg4510)	GSK2606414 (PERK inhibitor)	50 mg/kg BID	Oral gavage	6–7 wks	8–10	[17]
2	Halliday 2015	Prion disease	ISRIB (ISR inhibitor)	0.25 mg/kg	i.p., daily	≥4 wks	NR	[18]
3	Johnson 2016	AD (hAPP-J20)	ISRIB (inhibitor)	0.1; 0.25; 2.5 mg/kg	i.p. post-training	days	15/group	[19]
4	Bruch 2017	Tau (P301S)	PERK activator	2 mg/kg/day	i.p., daily	6 wks	NR	[20]
5	Krukowski 2020	Aging (19-mo)	ISRIB (inhibitor)	2.5 mg/kg	i.p., 3 doses	2–3 days	16–23	[21]
6	Sharma 2018	Hippocampal memory	GSK2606414 (PERK inhibitor)	Local infusion	CA1 infusion	days	NR	[22]
7	Koren 2021	Tauopathy (rTg4510)	GSK2606414 (inhibitor)	50–150 mg/kg	Oral	30 d	2–3 MRI cohorts	[23]
8	Ganz 2020	HD (R6/2)	MK-28 (PERK activator)	1 mg/kg	Osmotic pump	28 d	NR	[24]
9	Bai 2022	CMT1A/CMT1B	IFB-088/Sephin1 (ISR prolonger)	NR	Oral	weeks	NR	[25]
10	Das 2015	Misfolding disorders	Sephin1 (prolonger)	1 mg/kg	Oral/i.p.	weeks	NR	[26]
11	Chen 2019	CNS demyelination	Sephin1 (prolonger)	NR	NR	NR	NR	[27]
12	Hu 2022	AD (synaptic LTD)	ISRIB (inhibitor)	ex vivo/in vivo	Slice/rat paradigms	NR	NR	[28]

AD, Alzheimer’s disease; BID, twice daily; CA1, cornu Ammonis area 1; CMT, Charcot–Marie–Tooth disease; HD, Huntington’s disease; i.p., intraperitoneal; ISR, integrated stress response; ISRIB, integrated stress response inhibitor (eIF2B activator); MRI, magnetic resonance imaging; NR, not reported; PERK, PKR-like ER kinase; P301S/rTg4510, tauopathy mouse lines.

**Table 2:** Primary efficacy and biomarker outcomes.

#	Study	Primary Outcome(s)	Direction and Magnitude (If Numeric)	Target-Engagement Biomarkers	Ref.
1	Radford 2015	Neuronal survival, behavior in rTg4510	↓ neurodegeneration; improved function; numeric details NR	↓ PERK signaling; disease mitigation	[17]
2	Halliday 2015	Neurodegeneration and pancreatic toxicity readouts in prion	Prevented neurodegeneration without pancreatic toxicity	Partial restoration of protein synthesis; ↓ ATF4	[18]
3	Johnson 2016	MWM learning/memory in hAPP-J20	No rescue at 0.1–2.5 mg/kg; detailed stats reported	No ISR elevation; target engagement uncertain	[19]
4	Bruch 2017	Tau load and pathology (P301S)	PERK activation mitigated tau pathology; numeric NR	↑ eIF2α-P sustained	[20]
5	Krukowski 2020	RAWM and DMP cognition in aged mice	Improved spatial/working/episodic memory; ANOVA *p* < 0.05–0.01	↓ ATF4 and ↓ eIF2α in brain	[21]
6	Sharma 2018	Memory with local PERK blockade	Enhanced memory with CA1 PERK inhibition	Local kinase target engagement	[22]
7	Koren 2021	MEMRI-R1, brain atrophy, behavior	Ameliorated atrophy; functional improvements	Exposure 50–150 mg·kg^−1^ yields brain μM	[23]
8	Ganz 2020	Survival/toxicity in HD (R6/2)	MK-28 reduced toxicity; survival signals; numeric NR	PERK activation paradigm	[24]
9	Bai 2022	Motor/sensory neuropathy metrics	Improved neuropathy phenotypes	Prolonged ISR (GADD34-PP1)	[25]
10	Das 2015	Misfolding disease phenotypes	Prevented proteostasis-linked disease features	Selective PP1 regulatory subunit inhibition	[26]
11	Chen 2019	Oligodendrocyte/myelin preservation	Protection in demyelinating contexts	Prolonged ISR in CNS	[27]
12	Hu 2022	mGluR5-dependent LTD/synaptic function	Abrogated pathological LTD deficits	Functional ISR inhibition	[28]

ATF4, activating transcription factor 4; DMP, delayed matching-to-place; eIF2α, eukaryotic initiation factor 2 alpha; GADD34-PP1, protein phosphatase 1 complexed with PPP1R15A; LTD, long-term depression; MEMRI, manganese-enhanced MRI; MWM, Morris water maze; PERK, PKR-like ER kinase; RAWM, radial-arm water maze.

**Table 3:** Safety, exposure, and translational notes.

#	Study	Safety Signals	Brain Exposure/PK	Translational Notes	Ref.
1	Radford 2015	Systemic PERK inhibition associated with pancreatic toxicity at higher exposures	Effective CNS exposure with 50 mg/kg BID; details NR	Strong disease mitigation but safety limits systemics	[17]
2	Halliday 2015	No pancreatic toxicity with ISRIB	CNS target engagement evident	ISRIB safer vs. PERK inhibitors in prion model	[18]
3	Johnson 2016	No specific toxicity noted	Dose 0.1–2.5 mg/kg; exposure adequate	Model lacked ISR activation; highlight biomarker gating	[19]
4	Bruch 2017	No observable adverse events	NR	PERK activator viable in tau context	[20]
5	Krukowski 2020	Well-tolerated 2.5 mg/kg ISRIB	CNS engagement (↓ ATF4/↓ eIF2α)	Aging cognition—translationally relevant	[21]
6	Sharma 2018	Local infusion mitigates systemic toxicity	Local CA1 levels sufficient	Procedural targeting strategy	[22]
7	Koren 2021	Multikinase liabilities at high systemic exposure	50–150 mg/kg yields brain μM	Emphasizes off-target risk management	[23]
8	Ganz 2020	Continuous 1 mg/kg MK-28 tolerated	NR	Activating PERK strategy in HD	[24]
9	Bai 2022	Peripheral tolerability acceptable	NR	IFB-088 is clinically advancing	[25]
10	Das 2015	Favorable preclinical tolerability	NR	Seeding the IFB-088 translational path	[26]
11	Chen 2019	NR	NR	CNS demyelination support	[27]
12	Hu 2022	NR	NR	Synaptic physiology bridge to behavior	[28]

CNS, central nervous system; PK, pharmacokinetics; PERK, PKR-like ER kinase; µM, micromolar concentration (10^−6^ mol/L), reported here to indicate measured/estimated brain exposure levels; ATF4, activating transcription factor 4; NR, not reported.

**Table 4:** Outcome-direction matrix by ISR mechanism class across disease-model categories included in this review.

Disease-Model Category	eIF2B Activation	PERK Inhibition	PERK Activation	ISR Prolongation
Tauopathy/Alzheimer’s disease	Benefit in 3/4; 1/4 null (null reported in hAPP-J20 model); target engagement variably reported (ATF4 and/or p-eIF2α)	Benefit in 3/3; biomarker down-tuning of PERK-ISR signaling reported; safety limits for systemic PERK inhibition at higher exposures	Benefit in 1/1 (tau model) with sustained ISR signaling reported	—(not represented in included tau/AD entries)
Prion disease	Benefit in 1/1 with reported CNS protein-synthesis restoration; pancreatic toxicity not reported for ISRIB in included prion entry	—	—	—
Huntington’s disease	—	—	Benefit in 1/1 with survival/toxicity signals reported	—
Hereditary neuropathies (e.g., CMT models)	—	—	—	Benefit in 2/2 with motor/sensory phenotype improvement reported
CNS demyelination/leukodystrophy-type demyelination	—	—	—	Benefit in 1/1 with myelin/oligodendrocyte protection reported
Aging / age-related cognitive decline	Benefit in 1/1 with cognition improvement and brain biomarker shifts (ATF4 and/or p-eIF2α) reported	—	—	—

AD, Alzheimer’s disease; ATF4, activating transcription factor 4; CMT, Charcot–Marie–Tooth disease; CNS, central nervous system; eIF2B, eukaryotic initiation factor 2B; ISR, integrated stress response; ISRIB, integrated stress response inhibitor (eIF2B activator); PERK, PKR-like endoplasmic reticulum kinase.

## Data Availability

No new data were created or analyzed in this study.
